# A randomized controlled double-blind study on the brain protection of infantile patients with epileptic spasm syndrome through atomized inhalation of hydrogen-oxygen gas

**DOI:** 10.3389/fneur.2026.1781738

**Published:** 2026-03-12

**Authors:** Chen Chen, Wen He, Jian Chen, Xiu-Yu Shi, Guang Yang, Jing Wang, Yang-Yang Wang, Pei-Li Hu, Fang Han, Yan Meng, Li-Ping Zou

**Affiliations:** 1 Senior Department of Pediatrics, Chinese PLA General Hospital, Beijing, China; 2 Medical School of Chinese People’s Liberation Army, Beijing, China

**Keywords:** double-blind study, hydrogen-oxygen nebulization inhalation, infantile epileptic spastic syndrome, safety, therapeutic effect

## Abstract

**Objective:**

Hydrogen-oxygen nebulization inhalation has a brain-protective effect. This study aims to evaluate its short-term efficacy and safety as an adjuvant treatment for infantile epileptic spasms syndrome (IESS).

**Methods:**

A prospective randomized double-blind controlled trial enrolled 53 IESS children (Nov 2021–Nov 2023), randomized via a random number table into a hydrogen-oxygen inhalation group (27 cases, 66.6% H₂/33.3% O₂ nebulization) and an air inhalation group (26 cases, medical air nebulization). Both groups received standard treatment plus 4 daily 1-h interventions for 14 consecutive days. Primary endpoints were spasticity relief and effective rates; secondary endpoints included improvements in high-amplitude abnormal EEG, IL-6 levels, respiratory tract infection rate, and adverse reactions. Statistical analyses used SPSS software.

**Results:**

The primary efficacy endpoints (spasm relief rate *p* = 0.705, effective rate *p* = 0.950) and secondary efficacy endpoints (disappearance rate of electroencephalogram hypsarrhythmia *p* = 0.576, change in Kramer score *p* = 0.140, change in BASED score *p* = 0.168, abnormal IL-6 rate *p* = 0.081) of the two groups of children were compared. There was no statistically significant difference. The overall incidence of adverse reactions in the two groups was comparable, and no serious adverse events occurred in either group. However, the incidences of elevated myocardial enzymes (28.3% vs. 10.9%), gastrointestinal dysfunction (43.5% vs. 23.9%), and abnormal liver function (8.7% vs. 0%) in the hydrogen-oxygen inhalation group were significantly higher than those in the air inhalation group (all *p* < 0.05).

**Conclusion:**

Short-term hydrogen-oxygen nebulization inhalation failed to significantly improve the clinical efficacy of standard treatment for children with IESS. Although it is generally safe and feasible, its potential effects on myocardial, liver and gastrointestinal functions of children need to be closely monitored. In the future, large-sample, long-term follow-up multi-center studies need to be carried out to comprehensively evaluate its clinical value.

**Clinical trial registration:**

http://www.chictr.org.cn/bin/home, identifier (ChiCTR2100047479).

## Highlights

1. The first randomized double-blind controlled trial investigating the use of hydrogen and oxygen inhalation for the treatment of Infantile Epileptic Spasms Syndrome (IESS), focusing on this particularly vulnerable population of infants and young children.
2. Establish a comprehensive outcome evaluation system, covering spasticity relief, electroencephalogram (EEG) improvement, inflammatory cytokine levels, and multi-organ safety monitoring, with more systematic assessment dimensions.
3. It is clearly demonstrated that short-term hydrogen and oxygen inhalation does not show significant advantages in core efficacy indicators such as spasm control, while simultaneously revealing for the first time its specific adverse event risks in infant populations, providing critical references for clinical application.

## 1 Introduction

Infantile epileptic spasm syndrome (IESS) is one of the most common developmental epileptic encephalopathies in infancy and early childhood, belonging to the catastrophic epilepsy type, with an incidence rate of approximately 0.249 cases per 1,000 live births (1). The typical clinical trial is spastic seizure, hypsarrhythmia of electroencephalogram (EEG), and psychomotor retardation or regression. If not treated promptly, more than 80% of the children will develop severe psychomotor developmental disorders, and some may progress to more difficult-to-control epilepsy types such as Lennox–Gastaut syndrome, imposing a heavy burden on families and society (2). The core goal of protecting the developmental brain is to control seizures, reduce brain damage and preserve neural function.

Hydrogen is a promising neuroprotective agent and has become a research focus due to its powerful antioxidant properties. Hydrogen, as a small molecule substance with selective antioxidant and anti-inflammatory properties, can freely penetrate the blood–brain barrier and cell membranes, demonstrating potential therapeutic value in neurological diseases (3). Basic research has confirmed that hydrogen can exert neuroprotective effects in disease models such as cerebral ischemia and Parkinson’s disease through mechanisms like scavenging hydroxyl radicals, inhibiting the release of inflammatory factors, and reducing neuronal apoptosis (4, 5). A large number of studies have explored the potential applications of therapeutic gases in the treatment of various neurological diseases (6). In translational research on adult diseases, hydrogen has been proven to have neuroprotective effects in diseases such as cerebral ischemia and traumatic brain injury, and also plays a role in neurodegenerative diseases such as Alzheimer’s disease (7). Animal and human studies have verified the safety and feasibility of molecular hydrogen. However, despite a large number of studies on its therapeutic effects in adults. The translational animal model of neonatal hypoxic–ischemic encephalopathy (HIE) is the focus of studying the value of various gas therapies. Hydrogen ventilation, as a single dose or in combination with therapeutic hypothermia therapy, has demonstrated short-term and long-term neuroprotective effects in neonatal transforming HIE models (8). In animal experiments related to epilepsy, hydrogen has demonstrated clear anticonvulsant potential: pretreatment with inhaled hydrogen can significantly reduce the intensity of epileptic seizures, prevent neuronal loss, decrease the activation of microglia and astrocytes, and lower the levels of ROS, TNF-*α*, IL-1β, IL-6, CCL2 and CCL3. And increase the level of Nrf2. Inhaling hydrogen can also prevent the reduction of cerebral blood flow induced by kainic acid (KA). These results indicate that pretreatment with inhaled hydrogen can improve KA-induced epilepsy, inhibit inflammatory responses and oxidative stress, and thereby protect neurons (9). A study on asphyxiated newborn piglets demonstrated that the combination of therapeutic hypothermia intervention and hydrogen inhalation could significantly reduce the seizure burden within 24 h after hypoxic–ischemic injury, shorten the duration of status epilepticus, and improve the abnormal background manifestations of amplitude-integrated electroencephalogram (aEEG), with better effects than therapeutic hypothermia intervention alone. This study was the first to verify the anticonvulsant value of hydrogen in a neonatal mammalian epilepsy model, providing a key experimental basis for its clinical application (10). Given that IESS brain injury involves neuroinflammatory factor release (IL-6, TNF-*α*), oxidative stress-mediated apoptosis, and immature blood–brain barrier (BBB) permeability - while hydrogen can penetrate the BBB, scavenge hydroxyl radicals, and inhibit IL-6. Therefore, we hypothesize hydrogen-oxygen nebulization inhalation may provide targeted neuroprotective effect for IESS. As the world’s first randomized controlled double-blind trial based on positive results from animal experiments to convert hydrogen-oxygen nebulization inhalation for brain protection in patients with IESS, this study aims to clarify its short-term efficacy and safety as an adjunctive standard treatment. To provide new ideas for the treatment of children with epileptic encephalopathy and at the same time establish a translational research model from animal experiments to clinical research.

This study, as the world’s first randomized controlled double-blind trial of hydrogen-oxygen nebulization inhalation for the treatment of IESS, aims to clarify the short-term efficacy and safety of its adjuvant therapy and provide new ideas for the treatment of IESS.

## 2 Materials and methods

### 2.1 Research subjects

This study is a single-center, prospective, randomized, controlled, double-blind trial. The study protocol was approved by the Ethics Committee of our hospital in accordance with the Declaration of Helsinki (Ethics No.2021–188). All guardians signed informed consent forms, and the study was registered with the Chinese Clinical Trial Registry (Registration No.: ChiCRT2100047479). In accordance with relevant guidelines from the National Medical Products Administration (NMPA), particularly the requirements for medical device clinical trials and pediatric clinical trials, the standard operating procedures (SOP) of the study protocol detailed the procedures for hydrogen-oxygen nebulization inhalation, concentration control, monitoring frequency, and adverse event management. It included children with IESS admitted to Chinese PLA General Hospital from November 2021 to November 2023, with diagnostic criteria following the standards established by the International League Against Epilepsy (ILAE) in 2017. The diagnosis was jointly confirmed by 2–3 pediatric neurology experts.

#### 2.1.1 Inclusion criteria

(1) The age of onset was 2 months to 2 years;
(2) Meets the diagnostic criteria for IESS as defined by the International League Against Epilepsy (ILAE), confirmed by a pediatric neurologist;
(3) No adjustments were made to the children’s preexisting antiepileptic drug regimens during the 2 weeks before enrollment or throughout the trial.
(4) The guardian gave informed consent and signed the written informed consent form.

#### 2.1.2 Exclusion criteria

(1) The age of onset was less than 2 months or more than 2 years;
(2) Incomplete medical history;
(3) Combined with other serious diseases that are not suitable for participating in the trial;
(4) Participating in other medical trials;
(5) Enrolled children had their existing antiepileptic drug regimens adjusted during the 2 weeks prior to hydrogen-oxygen nebulization therapy and throughout the trial period.
(6) The guardian refuses to participate after being informed;
(7) Patients who had undergone epilepsy surgery for a clear etiology or were undergoing alternative therapies such as ketogenic diet or vagus nerve stimulation.

### 2.2 Research methods

#### 2.2.1 Grouping method

This study is a single center, randomized double-blind, controlled clinical trial. Using a random number table method, eligible children were divided into a hydrogen oxygen inhalation group and an air inhalation group. To ensure the effectiveness of the double-blind design, both groups used nebulization devices with identical appearance, sound, and operation methods, and the device numbers corresponded one-to-one with the patient numbers; All devices are managed and operated by dedicated nurses who have not participated in efficacy evaluation and follow-up. During the research process, both the researchers (including neurologists, evaluators) and the child guardians remained blinded to the grouping until all data collection was completed and unified unblinding was performed before statistical analysis. This design effectively avoids selection bias and evaluation bias, ensuring the scientific and reliable nature of research results.

#### 2.2.2 Treatment protocol

**Figure 1:**
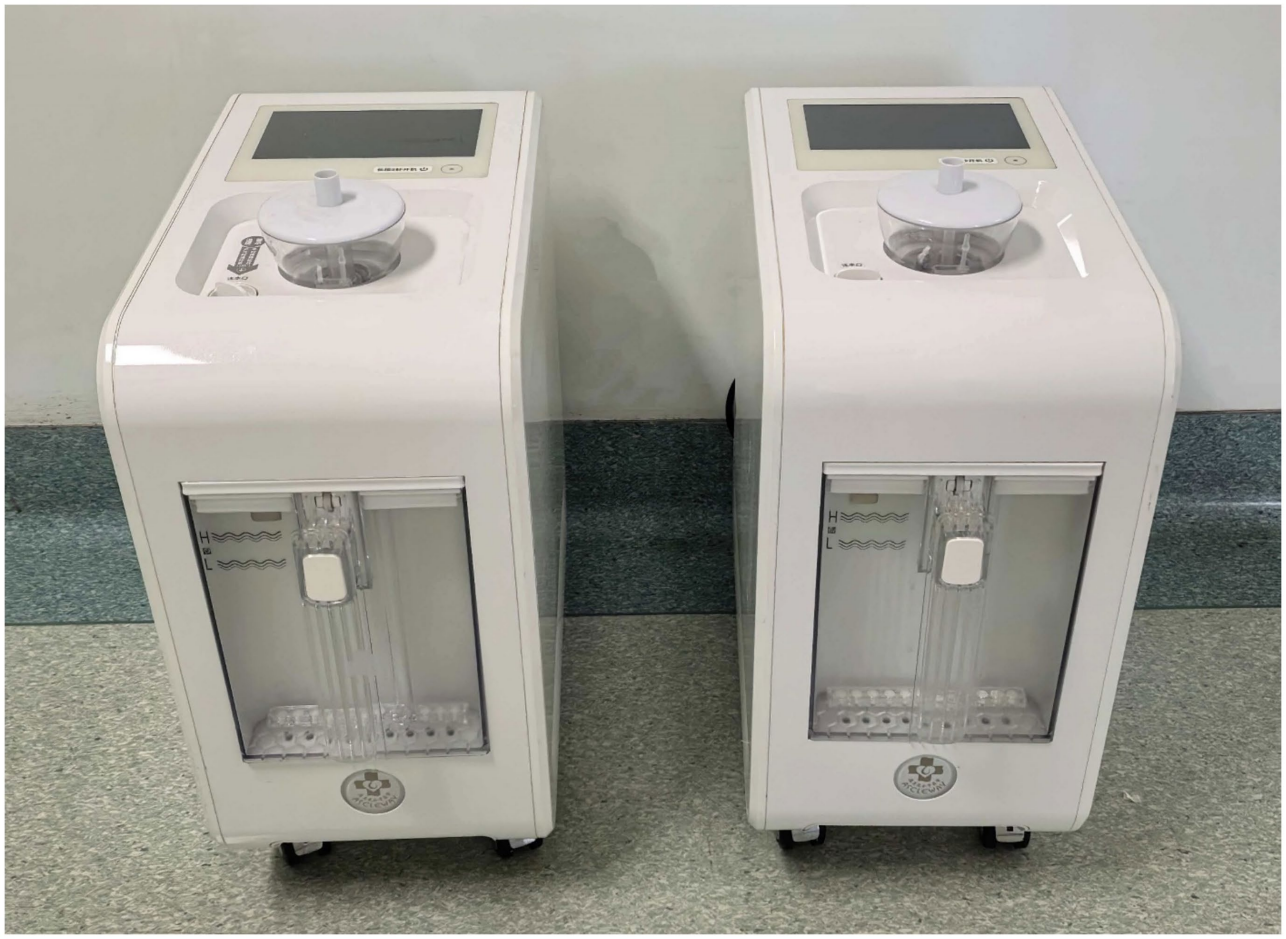
Appearance consistency between hydrogen-oxygen nebulization device (AMS-H-03) and air nebulization device. Both devices had identical operation/sound profiles, ensuring blinding.

Both groups of patients received IESS standard treatment (such as conventional treatment with antiepileptic drugs, etc.). On this basis, the hydrogen oxygen nebulization inhalation group uses the AMS-H-03 hydrogen oxygen generator produced by Shanghai Huimei Medical Technology Co., Ltd. to inhale a mixture of 66.6% hydrogen and 33.3% oxygen gas; The air nebulization inhalation group uses an air atomizer that has undergone special treatment by the company and has the same appearance as the hydrogen oxygen atomizer to inhale medical air (Figure 1: The appearance of the hydrogen oxygen nebulization inhalation machine is consistent with that of the air nebulization inhalation machine). Both groups were administered through a face mask with a flow rate of 3 L/min, inhaled for 1 h each time, 4 times a day, and intervened continuously for 14 days.

#### 2.2.3 Collection of clinical data

Collect baseline data from the children, including gender, age at admission, onset age, gestational age, mode of delivery, neonatal history, family history of neurological disorders, treatment intervals, Number of antiepileptic drugs (AEDs) used and prior ACTH therapy. Perform the following tests: ① Laboratory tests: complete blood count, liver and kidney function, cardiac enzyme profile, electrolytes, blood and urine metabolic screening, inflammatory factor (IL-6) detection, and genetic testing; ② Imaging studies: cranial CT, MRI, or PET-CT; ③ Neuroelectrophysiological tests: long-term video EEG monitoring.

#### 2.2.4 Efficacy and safety assessment

Efficacy and safety assessments were conducted before treatment and 14 days after treatment. Primary efficacy indicators included: ① Spasmodic remission rate: the proportion of cases with complete disappearance of spasmodic episodes after treatment; ② Response rate: the proportion of cases showing a reduction in spasmodic episode frequency by ≥50% after treatment. Secondary efficacy indicators comprised: ①EEG improvement: evaluation of EEG high-level disordered improvement using Kramer score and BASED score, with calculation of EEG high-level disordered disappearance rate; ② Inflammatory factor levels: detection and comparison of IL-6 abnormality rates before and after treatment in both groups; ③ Respiratory tract infection incidence.

Safety evaluation: The incidence of adverse reactions including elevated myocardial enzyme, electrolyte disorder, gastrointestinal dysfunction, rash, abnormal liver function, and restlessness were recorded through laboratory examination and clinical observation. The incidence of adverse reactions in the two groups was compared.

### 2.3 Statistical methods

Data analysis was performed using SPSS 26.0 statistical software. Quantitative data were presented as means ± standard deviations or medians (interquartile range) based on distribution characteristics. Group comparisons were conducted using independent samples t-test (for normal distribution) or Mann–Whitney U test (for non-normal distribution). Categorical data were expressed as frequencies (percentages), with χ^2^ test employed for intergroup comparisons. Fisher’s exact probability method was used when sample sizes were small. A *p* value ≤0.05 was considered statistically significant.

## 3 Results

### 3.1 Comparison of baseline data between the two groups

**Figure 2:**
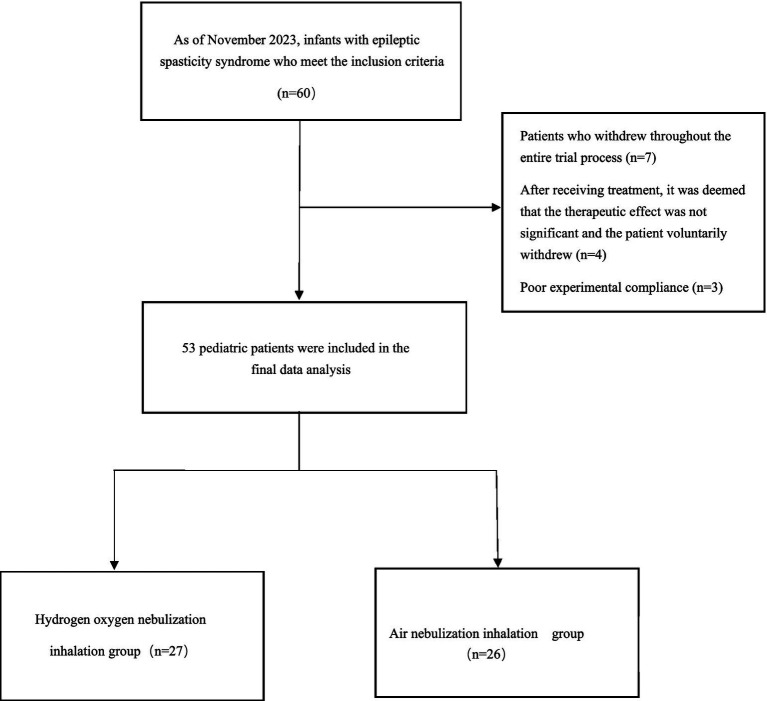
Flowchart of patient selection process.

A total of 60 eligible children were initially enrolled. Seven patients dropped out during the study, and 53 children completed treatment and were finally included in the efficacy analysis. For baseline characteristics that followed a normal distribution, data were presented as the mean ± standard deviation; for those with a non-normal distribution, data were expressed as the median (interquartile range) (Figure 2).

Hydrogen-oxygen nebulization inhalation group: 27 cases, 16 males (59.3%), 11 females (40.7%), male-to-female ratio of 1.45:1; height 76.6 ± 11.55 cm; median weight 9 kg. Median age at enrollment was 10 months, with the youngest being 2 months and the oldest 51 months. The onset age was 8.6 ± 5.93 months. Full-term infants: 23 cases (85.2%), preterm infants: 4 cases (14.8%); natural delivery: 12 cases (44.4%), cesarean delivery: 15 cases (55.6%); 10 cases (37.0%) had a history of neonatal diseases, including asphyxia, hypoxic–ischemic encephalopathy, pathological jaundice, hypoglycemia, purulent meningitis, leukoencephalopathy, intracranial hemorrhage, respiratory failure, bronchopulmonary dysplasia, etc. All children in the experimental group had no family history of neurological disorders.

No cases were identified with abnormal immune factors in the etiology classification. Among infectious causes, 2 cases (7.4%) had confirmed infections, specifically herpes simplex encephalitis and severe encephalitis. For metabolic causes, 23 cases (85.2%) underwent complete blood and urine metabolic screening, with 1 case (4.3%) showing reduced biotinase activity and no significant abnormalities in others. All cases underwent cranial imaging, revealing structural abnormalities in 10 cases (37.0%). Among these, 2 acquired structural abnormalities included severe encephalitis and herpes simplex viral encephalitis. Eight cases with unknown structural abnormalities included macrocephalic gyrus malformation, frontal lobe brain perforation malformation, and cerebellar and cerebral hemisphere atrophy.

In terms of genetic etiology, 20 cases underwent genetic testing, with 4 cases (20.0%) showing abnormalities. One case had a haplotype repeat of approximately 655Kbp in the q23 region of the X chromosome and a haplotype repeat of approximately 520Kbp in the q11.2 region of chromosome 5; one case had a heterozygous mutation in the SCN2A gene; one case had a heterozygous deletion of approximately 755Kbp in the q21.1q21.2 region of chromosome 1; and one case had a novel mutation in the NF1 gene. Fifteen cases (55.6%) had unknown etiology.

The median treatment interval was 3 months. Four cases (14.8%) had previously received ACTH therapy. Electroencephalogram (EEG) showed high dysrhythmia in 11 cases (40.7%) of the children. The pre-treatment Kramer score was 7.9 ± 2.15. The median pre-treatment BASED score was 4.0. Before treatment, 5 people (18.5%) had not taken AEDs, 10 people (37.0%) were on monotherapy, and 12 people (44.4%) were on polytherapy. The median baseline seizure frequency in the Hydrogen-oxygen nebulization inhalation group was 25 seizures/day.

Air nebulization inhalation group: 26 cases, 15 males (57.7%), 11 females (42.3%), male-to-female ratio of 1.36:1; median height is 75 cm; weight is 10.6 ± 2.55 kg. Median age at enrollment is 9 months, with the youngest being 3 months and the oldest 47 months; median age of onset is 5 months. Full-term infants: 24 cases (92.3%), preterm infants: 2 cases (7.7%); natural delivery: 13 cases (50.0%), cesarean delivery: 13 cases (50.0%); 4 cases (15.4%) have a history of neonatal diseases, mainly including hypoxic–ischemic encephalopathy, neonatal pneumonia, hypoglycemic encephalopathy, and pathological jaundice. 3 cases (11.5%) have a family history of neurological disorders.

In the etiology classification, no cases were identified with pathogenic factors caused by infectious or immune abnormalities. Regarding metabolic etiology, 25 children (96.2%) underwent complete blood and urine metabolic screening, among which 3 cases (12.0%) showed metabolic abnormalities: 1 case with mild ketoacidosis (VPA+), 1 case with elevated C3 level due to fatty acid metabolic defect, and 1 case with varying degrees of pyrimidine metabolites, 2,3-dihydroxy-2-methylbutyric acid, oxalate, and lysine elevation accompanied by ketonuria. For structural etiology, 26 children (100%) underwent cranial imaging examinations, with 18 cases (69.2%) demonstrating structural abnormalities. Among these, 3 acquired structural abnormalities included 2 cases of neonatal hypoglycemic encephalopathy and 1 case of neonatal hypoxic–ischemic encephalopathy. Genetic structural abnormalities included 1 new COL4A1 variant and 1 ARF1 gene heterozygous variant. Unexplained structural abnormalities (13 cases) comprised intracranial abnormality 3 signals, gray matter heterotaxy, and ventricular system enlargement. In genetic etiology, 20 children (76.9%) underwent genetic testing, with 7 cases (35.0%) showing abnormalities. Among the abnormal cases, 5 had confirmed pathogenic genes: 1 SYNGAP1 gene heterozygous variant, 1 DEPDC5 gene heterozygous mutation, 1 ARF1 gene heterozygous variant, 1 SZT2 gene heterozygous variant, and 1 new COL4A1 variant. The other 2 cases were suspected pathogenic genes: 1 CACNA1H gene heterozygous variant and 1 CACN1H gene heterozygous variant. Unexplained cases accounted for 16 children (61.5%).

The median treatment interval was 3.5 months, with 10 patients (38.5%) having received ACTH therapy. Electroencephalogram (EEG) showed high dysrhythmia in 13 cases (50%). The median pre-treatment Kramer score was 8, with 11 cases (42.3%) ≤ 8 and 15 cases (57.7%) > 8. The median pre-treatment BASED score was 4. Before treatment, 0 patients (0.0%) had not taken AEDs, 8 patients (30.8%) were on monotherapy, and 18 patients (69.2%) were on polytherapy. The air nebulization inhalation group had a baseline seizure frequency of 27.1 episodes per day.

The baseline characteristics between the two groups were analyzed, with specific results presented in Table 1 (Comparison of baseline characteristics between the hydrogen-oxygen nebulization group and air nebulization group). There were no statistically significant differences between the two groups in terms of gender (*p* = 0.90), age at enrollment (*p* = 0.69), age at onset (*p* = 0.07), treatment interval (*p* = 0.73), gestational age (*p* = 0.41), mode of delivery (*p* = 0.69), neonatal disease history (*p* = 0.14), family history of neurological disorders (*p* = 0.07), etiology classification (*p* = 0.60), EEG hypsarrhythmia (*p* = 0.50), EEG Kramer scores and grading (*p* = 0.37), EEG BASED scores and grading (*p* = 0.38), number of antiepileptic drugs used (*p* = 0.12), ACTH treatment history (*p* = 0.10), and baseline seizure frequency (*p* = 0.62).

**Table 1:** Comparison of baseline characteristics between the hydrogen-oxygen nebulization group and the air nebulization group.

Baseline features	Hydrogen-oxygen nebulization inhalation group (*n* = 27)	Air nebulization inhalation group (*n* = 26)	P price
Sex			0.90
Man	16 (59.3%)	15 (57.7%)	
Woman	11 (40.7%)	11 (42.3%)	
Height (cm)	76.6 ± 11.55	75 (71.5, 80.0)	0.75
Weight (kg)	9 (8.0, 10.0)	10.6 ± 2.55	0.22
Group age (months)	10 (6, 13)	9 (6, 13)	0.69
Age at onset (months)	8.6 ± 5.93	5 (3, 8)	0.07
Interval of treatment (month)	3 (2, 5)	3.5 (2, 6)	0.73
Gestational age			0.41
Term infant	23 (85.2%)	24 (92.3%)	
Premature	4 (14.8%)	2 (7.7%)	
Mode of production			0.69
Natural labor	12 (44.4%)	13 (50.0%)	
C-sect	15 (55.6%)	13 (50.0%)	
History of neonatal disease	10 (37.0%)	4 (15.4%)	0.14
Family history of neurological disease	0 (0%)	3 (11.5%)	0.07
Etiology			0.60
Heredity	4 (14.8%)	7 (26.9%)	
Metabolic	1 (3.7%)	3 (11.5%)	
Constitutive property	10 (37.0%)	18 (69.2%)	
Infectious	2 (7.4%)	0 (0%)	
Immunity	0 (0%)	0 (0%)	
Unknown	10 (37.0%)	5 (19.2%)	
Highly disordered EEG	11 (40.7%)	13 (50.0%)	0.50
Kramer Score	7.9 ± 2.15	8 (7, 9)	0.37
BASED Score	4.0 (3.0, 5.0)	4.0 (3.0, 5.0)	0.38
Number of AEDs used			0.12
Not medicated	5(18.5%)	0(0.0%)	
Monotherapy	10(37.0%)	8(30.8%)	
Multiple drugs	12(44.4%)	18(69.2%)	
Baseline seizure frequency (times/day)	25 (11, 52.5)	27.1 (15, 56.13)	0.62
Previous ACTH treatment history	4 (14.8%)	10 (38.5%)	0.10

Parameter description. Gender: Male = Male; Female = Female. Height (cm): Continuous variable, expressed as mean ± standard deviation. Weight (kg): Continuous variable, expressed as median (interquartile range). Group age (months): continuous variable, expressed as median (interquartile range). Age of onset (months): continuous variable, expressed as mean ± standard deviation. Treatment interval (months): The time from initial diagnosis to enrollment, expressed as the median (interquartile range). Gestational age: full-term infants = ≥ 37 weeks; Premature infants = <37 weeks. Delivery method: natural delivery vs cesarean section. History of neonatal diseases: Refers to the presence of asphyxia, hypoxic–ischemic encephalopathy, intracranial hemorrhage, etc. within one month after birth. Neurological family history: Refers to neurological disorders such as epilepsy, intellectual disability, cerebral palsy, etc. in direct relatives.

Etiological classification. Hereditary: related to gene mutations (such as SCN1A, CDKL5, etc.). Metabolic factors: such as organic acidosis, mitochondrial disease, etc. Structural: brain developmental abnormalities, cortical dysplasia, etc. Infectious: Perinatal infection leading to brain damage. Immunological: Autoimmune encephalitis, etc. Unknown: those with an unclear cause. EEG high arrhythmia: Did the EEG display a typical high arrhythmia pattern at the time of enrollment. Kramer score: evaluates the neurological development status of infants, ranging from 0 to 12 points, with higher scores indicating poorer development. BASE Score: based on the severity of epilepsy syndrome according to clinical manifestations (grades 1–5), the larger the number, the more severe the condition. Number of AEDs used: the number of antiepileptic drugs taken before treatment. Patients were classified into three categories: not medication, monotherapy and multiple drugs. Baseline seizure frequency (times/day): The average daily frequency of spastic seizures in the 7 days prior to enrollment, expressed as the median (interquartile range). Have you received ACTH treatment: Refers to the use of adrenocorticotropic hormone to treat IESS before the start of this study.

### 3.2 Short-term efficacy of hydrogen and oxygen nebulization inhalation in infantile epileptic spasms syndrome

#### 3.2.1 Comparison of primary outcomes between the hydrogen-oxygen nebulization inhalation group and the air nebulization inhalation group

##### 3.2.1.1 Reduction in the frequency of convulsive episodes

The median reduction rate of convulsive seizures on the day of completion of hydrogen‑oxygen nebulization therapy was 91.11% (55.88%, 100%), which was lower than the 100% reduction rate (61.47, 100%) observed in the air nebulization group. Mann–Whitney U test showed *p* = 0.705, indicating that although the reduction rate in the hydrogen-oxygen nebulization group was lower than that in the air nebulization group, the difference did not reach statistical significance (Table 2).

**Table 2:** Comparison of primary outcomes between hydrogen-oxygen nebulization group and air nebulization group.

Primary outcome measures	Hydrogen-oxygen nebulization inhalation group (*n* = 27)	Air nebulization inhalation group (*n* = 26)	P price
Reduction rate of seizure attacks			0.705
Median (IQR)	91.11% (55.88, 100%)	100% (61.47,100%)	
Spasm attack treatment effectiveness rate			0.950
Valid, *n*(%)	23 (85.19%)	21 (80.77%)	
Invalid, *n* (%)	4 (14.81%)	5 (19.23%)	

Parameter description. Spasmodic seizure reduction rate: refers to the percentage of decrease in the average daily number of spastic seizures after treatment compared to the baseline period.

Effective treatment rate: according to the International League Against Epilepsy (ILAE) standard definition, a reduction of ≥ 50% in spastic seizures is considered “effective,” and<50% is considered “ineffective”.

##### 3.2.1.2 Effective rate of convulsive seizure treatment

The number of effective patients in the hydrogen-oxygen nebulization inhalation group was 23 (85.19%), and the number of ineffective patients was 4 (14.81%). The number of effective patients in the air nebulization inhalation group was 21 (77.78%), and the number of ineffective patients was 5 (18.52%). The *p* value of the continuous modified chi-square test was 0.950, and there was no statistical difference between the two groups (Table 2).

#### 3.2.2 Comparison of secondary outcomes between hydrogen and oxygen nebulization inhalation groups and air nebulization inhalation group

##### 3.2.2.1 Influence of ACTH treatment history on efficacy

An intergroup comparison was conducted on ACTH treatment history between the two groups. In the hydrogen-oxygen group, 23 patients received their first ACTH treatment, with a 96.67% reduction rate in seizure episodes (70%, 100%). The air group had 16 patients receiving their first ACTH treatment, achieving a 100% reduction rate (70%, 100%). The Mann–Whitney U test showed no statistically significant difference (*p* = 0.767). For the second ACTH treatment, 4 patients in the hydrogen-oxygen group achieved a 67.80% ± 29.26% reduction rate, while 10 patients in the air group recorded a 98.485% reduction rate (38.160%, 100%). The Mann–Whitney U test again indicated no statistically significant difference (*p* = 0.635).

An intra-group comparison was conducted on ACTH treatment history between the two groups. In the Hydrogen Oxygen group, 23 patients received their first ACTH treatment, with a seizure reduction rate of 96.67% (70%, 100%). For the second ACTH treatment, 4 patients in the Hydrogen Oxygen group showed a seizure reduction rate of 67.80% ± 29.26%. The Mann–Whitney U test revealed no statistically significant difference between the groups (*p* = 0.409). In the Air group, 16 patients received their first ACTH treatment, achieving a 100% seizure reduction rate (65.20%, 100%). For the second ACTH treatment, 10 patients in the Air group demonstrated a 98.485% seizure reduction rate (38.160%, 100%). The Mann–Whitney U test again showed no statistically significant difference between the groups (*p* = 0.660). Therefore, we conclude that the frequency of ACTH treatment has no significant impact on the efficacy of Hydrogen Oxygen nebulization therapy, whether compared between groups or within groups (Table 2).

##### 3.2.2.2 Comparison of the degree of brainwave irregularity between the two groups

The clinical outcome was assessed by the disappearance of high-amplitude EEG disorganization. Among 11 children with pre-treatment high-amplitude EEG disorganization, 9 cases (81.8%) showed complete resolution after hydrogen-oxygen nebulization inhalation therapy. In the air inhalation group, 12 out of 13 children (92.3%) achieved this resolution. Fisher’s exact test revealed no statistically significant difference between the two groups (*p* = 0.576).

In order to evaluate the post-treatment EEG indicators from more perspectives and objectively, we used both Kramer scoring scale and BASED scoring scale to score the EEG of each child before and after treatment.

Using the EEG Kramer scoring scale as the evaluation criterion, both groups showed significant decreases in scores. The Kramer scores before and after treatment were 7.93 ± 2.147 and 7 (4,7) in the hydrogen-oxygen nebulization group (*p* = 0.001), respectively, while those in the air nebulization group were 8.73 ± 2.127 and 5.69 ± 2.055 (*p* < 0.001). These results indicate that the EEG hypsarrhythmia in IESS patients was significantly improved after treatment in this trial. However, the mean reduction in Kramer scores between the two groups was 2 ± 3 and 3 ± 2 (*p* = 0.140), respectively, showing no statistically significant difference.

Using EEG BASED scoring scale as the evaluation criterion, both groups showed significant decreases. The hydrogen-oxygen nebulization inhalation group recorded BASED scores of 4 (3,5) and 3 (3,4) pre-and post-treatment (*p* < 0.001), respectively, while the air atomized inhalation group showed scores of 4 (4,5) and 3 (3,4) (*p* < 0.001). These results indicate marked improvement in EEG hypsarrhythmia among IESS patients after treatment. However, the mean BASED score reductions were 1 (0,1) and 1 (0,1) (*p* = 0.168) in both groups, showing no statistically significant difference (Table 3).

**Table 3:** Comparison of secondary outcomes between hydrogen-oxygen nebulization group and air nebulization group.

Secondary outcome measures	Hydrogen-oxygen nebulization inhalation group (*n* = 27)	Air nebulization inhalation group (*n* = 26)	P price
ACTH treatment history			
Reduction rate of seizure episodes in first ACTH administration	96.67% (70, 100%)	100% (65.20,100%)	0.767
Reduction rate of seizure episodes in patients receiving ACTH for the second time	67.80% ± 29.26%	98.485%(38.160,100%)	0.635
Highly disordered EEG disappearance rate			0.576
Number of children with high dysrhythmia before treatment	11	13	
Number of children with disappearance of high dysrhythmia after treatment	9 (81.8%)	12 (92.3%)	
Kramer score change			0.140
Pre-treatment score	7.93 ± 2.147	8.73 ± 2.127	
Post-treatment score	7 (4, 7)	5.69 ± 2.055	
Average decrease	2 ± 3	3 ± 2	
BASED score change			0.168
Pre-treatment score	4 (3, 5)	4 (4, 5)	
Post-treatment score	3 (3, 4)	3 (3, 4)	
Average decrease	1 (0, 1)	1 (0, 1)	
Respiratory tract infection rate during treatment	4 (14.8%)	0 (0%)	0.111
Abnormal rate of interleukin-6 (IL-6)			0.081
Pre-treatment abnormality rate	1/24 (4.2%)	3/24 (12.5%)	
Post-treatment abnormality rate	1/23 (4.3%)	5/20 (25.0%)	

Parameter description. The reduction rate of seizures after the first ACTH treatment: refers to the percentage of children who received ACTH treatment before the start of the study who experienced a decrease in the number of spastic seizures after the first use of ACTH compared to baseline, expressed as the median (interquartile range); Reduction rate of seizures after the second ACTH treatment: refers to the reduction rate of seizures after the second medication in children who have received ACTH treatment twice. The hydrogen oxygen group is expressed as mean ± standard deviation, while the air group is expressed as median (IQR) due to non normal distribution of data. The disappearance rate of highly irregular EEG: refers to the proportion of children with highly irregular EEG before treatment who have normal or significantly improved EEG after treatment. Kramer score change: used to evaluate neurological development status, ranging from 0 to 12 points, with higher scores indicating poorer development. BASED score changes: based on clinical manifestations, the severity of epilepsy syndrome is graded (1–5 levels), with larger numbers indicating more severe symptoms. The incidence of respiratory infections during treatment: refers to the number of cases with respiratory infection symptoms such as fever, cough, and sputum that occur during the intervention period and are confirmed by doctors, expressed in frequency (percentage); Fisher’s exact test was used for inter group comparison. Abnormal rate of interleukin-6 (IL-6): refers to the proportion of children with serum IL-6 levels higher than the laboratory upper limit of normal.

##### 3.2.2.3 Rate of respiratory tract infection in the two groups during treatment

From the first day of ACTH treatment to the final day, The hydrogen-oxygen nebulization inhalation group reported 4 cases of respiratory tract infections (RTIs) during treatment, with an RTI rate of 14.8%. In contrast, the air nebulization inhalation group showed no RTIs in all patients, achieving a 0% infection rate. Fisher’s exact test (*p* = 0.111) indicated no statistically significant difference between the two groups (Table 3).

##### 3.2.2.4 Abnormal rate of IL-6 detection

The intra-group comparison was made between the group of hydrogen and oxygen mixed nebulization inhalation and the group of air nebulization inhalation before and after treatment.

Before treatment, 24 children (88.9%) in The hydrogen-oxygen nebulization inhalation group underwent interleukin-6 (IL-6) testing, while 3 children (11.1%) did not. Among the tested group, 23 children (95.8%) had normal IL-6 levels, with 1 case showing abnormality (4.2%). After treatment, 23 children (85.2%) in the HPC group were tested, and 4 children (14.8%) were excluded. The tested group showed 22 children (95.7%) with normal IL-6 levels and 1 abnormal case (4.3%). Fisher’s exact test indicated *p* = 1.000, indicating no statistically significant difference in IL-6 abnormality rates between the treatment groups before and after therapy.

Before treatment, 24 children (92.3%) in the air nebulization group underwent interleukin-6 (IL-6) testing, while 2 children (7.7%) did not. Among the tested children, 21 (87.5%) had normal IL-6 levels, and 3 (12.5%) showed abnormalities. After treatment, 20 children (76.9%) in the air nebulization group underwent IL-6 testing, and 6 children (23.1%) did not. Among the tested children, 15 (75%) had normal IL-6 levels, and 5 (25%) showed abnormalities. The Fisher exact test revealed a *p*-value of 0.436, indicating no statistically significant difference in the abnormality rate of IL-6 between the pre-and post-treatment groups in the air nebulization group.

The interleukin-6 (IL-6) levels were compared between the hydrogen-oxygen mixed nebulization inhalation group and the air nebulization inhalation group post-treatment. Fisher’s exact test showed *p* = 0.081, indicating no statistically significant difference in the abnormal rate of IL-6 levels between the two groups (Table 3).

##### 3.2.2.5 Number of days hospitalized and treatment costs

The average hospitalization duration in the hydrogen-oxygen nebulization inhalation group was 28.41 ± 8.154 days, with treatment costs amounting to 30,312.51 (28,001.81, 34,357.37) RMB.

The hospitalization duration in the air nebulization inhalation group was 25 (19.75,29) days, with treatment costs amounting to 30,827.385 (22,496.3225,35,566.0050) RMB.

The comparison of the two groups of hospitalization days *p* = 0.103, the two groups of treatment costs *p* = 0.477, there is no significant difference.

### 3.3 Safety analysis of hydrogen and oxygen nebulization inhalation

Among all 53 enrolled children, 46 cases (86.8%) had adverse reactions of different degrees during treatment. There were 25 cases (54.3%) in the hydroxyl oxygen nebulization inhalation group and 21 cases (45.7%) in the air nebulization inhalation group. The difference of adverse reactions between the two groups was not significant (*p* = 0.204).

None of the adverse reactions occurred in children with severe or life-threatening manifestations. Among the 7 participants who discontinued treatment, 4 withdrew due to personal reasons after treatment initiation, while 3 discontinued due to poor trial adherence. In the hydrogen-oxygen nebulization group, 13 cases (28.3%) showed elevated cardiac enzymes, 9 (19.6%) had hypokalemia, 20 (43.5%) experienced gastrointestinal dysfunction, 5 (10.9%) developed rashes, 4 (8.7%) showed liver function abnormalities, and 2 (4.3%) exhibited irritability. The air nebulization group reported 5 cases (10.9%) of elevated cardiac enzymes, 6 (13.0%) of hypokalemia, 11 (23.9%) of gastrointestinal dysfunction, 7 (15.2%) of rashes, and 1 (2.2%) of irritability, with no liver function abnormalities observed in this group. Chi-square analysis revealed statistically significant differences between groups in cardiac enzyme elevation (*p* = 0.026), gastrointestinal dysfunction (*p* = 0.019), and liver function abnormalities (*p* = 0.041), while other adverse reactions showed no significant differences. All 4 patients who developed liver function abnormalities received standard hepatoprotective treatment. Among them, liver function parameters returned to normal on the day the trial ended in 2 cases, while the other 2 cases recovered within one week after the completion of the trial, with no residual liver damage or other sequelae observed.

## 4 Discussion

IESS, a severe developmental epileptic encephalopathy in infancy, has a complex pathogenesis involving genetic, metabolic, and structural factors. Neuroinflammatory responses, oxidative stress, and mitochondrial dysfunction play pivotal roles in disease progression (11). Elevated levels of inflammatory factors such as IL-1β, TNF-*α*, and IL-6 can disrupt the blood–brain barrier, exacerbating abnormal neuronal excitability. Concurrently, dysregulation of GABAergic neurotransmitter modulation further leads to excitatory imbalance in brain networks, manifesting as typical electroencephalographic (EEG) hyperactivity and convulsive seizures (12, 13). Current clinical treatments struggle to simultaneously achieve efficient seizure control and neuroprotective effects, thereby failing to ensure favorable neurodevelopmental outcomes.

Hydrogen, as a novel medical gas, exhibits characteristics such as selective scavenging of hydroxyl radicals, inhibition of inflammatory responses, and reduction of neuronal apoptosis. Its small molecular structure enables it to freely penetrate the blood–brain barrier, demonstrating unique advantages in neurological disorders (14, 15). Basic research has confirmed that hydrogen exerts neuroprotective effects in models of cerebral ischemia and traumatic brain injury by modulating signaling pathways such as NF-κB and Nrf2 (16–18). In animal models of epilepsy, hydrogen can reduce seizure intensity and shorten seizure duration (19, 20). This study, as the world’s first randomized controlled double-blind clinical trial applying hydrogen-oxygen intervention to IESS, not only adopts a non-invasive nebulization inhalation method suitable for infants and young children but also systematically designs and validates the clinical protocol based on prior animal experiments (e.g., improving aEEG background and shortening status epilepticus in hypoxic–ischemic brain injury models with H2 combined with hypothermia). It successfully establishes a complete translational chain from “animal experiments to clinical research.”

The primary endpoints (spasm relief rate and response rate) directly correspond to core symptoms of IESS, while secondary endpoints (EEG hypsarrhythmia resolution rate, Kramer/BASED score, and IL-6 levels) reflect neuroelectrophysiological (EEG abnormalities) and inflammatory mechanisms (IL-6). Although short-term hydrogen-oxygen therapy did not significantly enhance efficacy, the multidimensional evaluation system comprehensively reflected IESS disease characteristics, aligning with hydrogen’s fundamental mechanisms of “anti-inflammatory (inhibiting IL-6) and antioxidant (improving neuronal damage)” (21). Through systematic safety monitoring, this study for the first time delineated the overall safety profile and organ risk boundaries of hydrogen-oxygen therapy in infantile IESS populations. Simultaneously, multidimensional efficacy indicators strongly corresponded with established mechanisms, providing critical reference for optimizing intervention parameters (e.g., extended treatment duration, concentration adjustments) and exploring combination strategies with standard therapies like ACTH.

Short-term hydrogen-oxygen nebulization inhalation failed to significantly enhance the efficacy of IESS standard treatment, with no statistically significant differences observed between the two groups in core indicators such as seizure relief rate, response rate, and EEG improvement. This result contradicts expectations and may be attributed to the following reasons: ① Short intervention duration: The 14-day intervention may be below hydrogen’s neuroprotective threshold. Previous HIE animal models required >21 days, while adult stroke studies used 4-week courses. As IESS is a chronic refractory disease, a 14-day short-term intervention may not be sufficient for hydrogen’s neuroprotective effects to fully manifest, and seizure control and EEG improvement often require longer periods. If the intervention period is extended to 3–4 weeks, and higher hydrogen concentrations (such as 80%) are attempted or the daily treatment frequency is increased (for example, six times a day), the effectiveness may be improved. The results of this study provide important reference for future optimization of hydrogen-oxygen nebulization inhalation therapy for IESS; ② Limitations in biomarker monitoring: The study only measured the single inflammatory factor IL-6, whereas hydrogen’s anti-inflammatory effects may involve multiple cytokine networks, making it difficult for a single biomarker to comprehensively reflect its therapeutic effects.

The primary concern in this study is the safety of hydrogen-oxygen nebulization inhalation (hydrogen concentration 66.7%, oxygen concentration 33.3%), especially in infants under 1 year of age. Currently, there are relevant animal studies (including neonatal mice) and adult clinical studies on this concentration of hydrogen-oxygen mixture, which can provide strong support for this study.

Existing animal studies have confirmed the safety and efficacy of this concentration of hydrogen-oxygen mixed gas. Among them, studies related to neonatal mice have focused on neuroprotection and organ injury repair. For example, research on neonatal hypoxic–ischemic encephalopathy (HIE) in mice has shown that inhalation of a 66.7% hydrogen/33.3% oxygen mixed gas aerosol can reduce brain tissue damage in neonatal mice and improve neurobehavioral outcomes by clearing hydroxyl radicals and inhibiting neuroinflammatory responses. During the intervention, no adverse reactions such as respiratory distress, arrhythmia, or abnormal weight were observed in the neonatal mice, confirming its good safety in neonatal mice (22). This study is a real-world investigation on hydrogen-oxygen inhalation in neonatal mice with HIE, focusing on neuroprotective effects, which is highly consistent with the direction of this research and provides core evidence for it. Existing studies have confirmed that a 66.7% hydrogen/33.3% oxygen gas mixture is a commonly used safe concentration in hydrogen-oxygen medical research. Its physical and chemical properties are stable, and it shows no significant toxicity (23). It should be noted that research on inhalation of hydrogen-oxygen mixtures at concentrations of 66.7%/33.3% for infantile epileptic spasms syndrome (IESS) is still relatively scarce. This study can provide additional safety and efficacy data for the infant population in this field.

During the entire intervention period, we established a comprehensive safety monitoring framework to monitor adverse reactions, including daily vital signs monitoring, regular blood and biochemical tests (liver and kidney function, electrolytes, blood gas analysis), and proactive reporting of adverse events (AEs). We focused on sensitive organs in infants: specifically monitoring the sensitive functions of the myocardium, liver, and gastrointestinal tract. It was found that although the hydrogen-oxygen group was generally well tolerated, the incidences of elevated myocardial enzymes (28.3% vs. 10.9%), gastrointestinal dysfunction (43.5% vs. 23.9%), and abnormal liver function (8.7% vs. 0%) were significantly higher than those in the control group (*p* < 0.05). This finding has been rarely reported in hydrogen therapy for other diseases and clearly identifies the organ safety boundaries that need particular attention in the infant population, providing key data support for monitoring protocols during clinical application (such as regular reexamination of myocardial enzymes and liver function). However, there was no statistically significant difference in the incidence of adverse events between the two groups, and the vast majority were mild to moderate, with no clear causal relationship to hydrogen-oxygen nebulization inhalation. This confirms that hydrogen-oxygen nebulization inhalation is generally well tolerated, but monitoring of the above organ functions should be strengthened during clinical use.

This study has certain limitations: the single-center design and small sample size result in insufficient statistical power, making it difficult to detect subtle therapeutic differences, and the conclusions are limited in extrapolation. The intervention parameters were fixed, and no dose–response relationship studies were conducted, leaving the optimal hydrogen-oxygen concentration, inhalation duration, and treatment course for IESS therapy unclear. The follow-up period was short, with only short-term efficacy assessed and no attention paid to long-term neurodevelopmental outcomes. Mechanistic exploration was inadequate, as no in-depth research was performed using cerebrospinal fluid oxidative stress markers or brain functional imaging. Future studies could improve in the following aspects: conducting large-sample, multicenter randomized controlled trials to increase statistical power and expand the sample size, while incorporating different etiological subgroups for stratified analysis; optimizing the intervention protocol to explore the effects of different hydrogen-oxygen concentrations, inhalation durations, and treatment courses on efficacy and identify optimal therapeutic parameters; extending the follow-up period and using tools such as the Bayley Infant Developmental Scale to assess long-term neurodevelopmental outcomes; and integrating multidimensional biomarker testing (e.g., multiple inflammatory factors, oxidative stress markers) and brain functional imaging techniques to further investigate the mechanisms of hydrogen-oxygen therapy.

## 5 Conclusion

The 14-day short-term hydrogen-oxygen nebulization inhalation as adjuvant therapy for Infantile Epileptic Spasms Syndrome (IESS) failed to significantly enhance the clinical efficacy of standard treatment. Although the therapy was generally safe and feasible, the hydrogen-oxygen nebulization inhalation group exhibited significantly higher rates of myocardial enzyme elevation, gastrointestinal dysfunction, and hepatic abnormalities. Close monitoring of organ functions is essential during clinical application. Future studies should conduct large-scale, multicenter, long-term follow-up clinical trials and optimize intervention protocols to comprehensively evaluate the clinical value of hydrogen-oxygen therapy for IESS.

## Data Availability

The original contributions presented in the study are included in the article/supplementary material, further inquiries can be directed to the corresponding author.
